# Incidence and characteristics of ventricular tachycardia in patients after percutaneous coronary revascularization of chronic total occlusions

**DOI:** 10.1371/journal.pone.0225580

**Published:** 2019-11-22

**Authors:** Sebastian König, Enno Boudriot, Arash Arya, Julia-Anna Lurz, Marcus Sandri, Sandra Erbs, Holger Thiele, Gerhard Hindricks, Borislav Dinov

**Affiliations:** 1 Department of Electrophysiology, Heart Center Leipzig at University of Leipzig, Leipzig, Sachsen, Germany; 2 Department of Cardiology, Heart Center Leipzig at University of Leipzig, Leipzig, Sachsen, Germany; 3 Leipzig Heart Institute, Leipzig, Sachsen, Germany; Klinikum Region Hannover GmbH, GERMANY

## Abstract

**Objectives:**

This study sought to investigate the prevalence of ventricular tachycardia after percutaneous coronary intervention (PCI) of chronic total occlusion (CTO).

**Background:**

PCI of a CTO is associated with improvement of the left ventricular ejection fraction and possibly associated with reduced mortality. However, benefits of CTO-PCI must be weighed against a higher risk of procedure-related complications. The incidence of new-onset ventricular tachycardia after a successful CTO-PCI has not been investigated so far. In this retrospective registry we seek to describe characteristics and predictors of occurrence of post-procedural ventricular tachycardias.

**Methods and results:**

Between 2010 and 2015, 485 patients underwent successful CTO-PCI at Heart Center Leipzig. Of them, 342 had complete follow-up and were further analyzed. Ventricular tachycardias were detected in 9 (2.6%) patients. All of them were monomorphic ventricular tachycardias occurring in median 1 day (interquartile range [IQR] 0.25–4.75 days) after PCI and caused prolongation of the hospital stay. Patients with ventricular tachycardia were older, had worse left ventricular ejection fraction (mean 33.1%, SD 5.9%) and more frequently a CTO of an infarct-related artery. The target vessel was not associated with the occurrence of ventricular arrhythmias. In multivariable analysis, only impaired left ventricular systolic function was an independent predictor for procedure-related ventricular tachycardia. Mortality rates were not different between patients with or without ventricular tachycardia.

**Conclusion:**

Ventricular tachycardia can occur early after CTO-PCI as possible reperfusion arrhythmia and poorer left ventricular ejection fraction is the only independent predictor for onset. Although the occurrence of ventricular tachycardia after CTO-PCI seems not to influence mortality, awareness of this possible complication and longer monitoring may be recommended.

## Introduction

Percutaneous coronary intervention (PCI) for revascularization of a chronic total occlusion (CTO) is a challenging procedure. Nevertheless, the field is expanding driven by the high prevalence of CTO, technical improvements, rising success rates, symptom relief and a possible survival benefit in various patient cohorts.[1, 2] A CTO usually is defined as a total occlusion of a coronary artery for more than three months with resulting TIMI grade 0–1 flow.[3] CTOs are common findings in coronary angiography with a prevalence of up to 18.6% of all patients referred for coronary angiography. Furthermore, 18–52% of all patients with significant coronary artery disease (CAD) have at least one CTO.[4–6] Patients with CTOs more often have diabetes and left ventricular dysfunction than patients with CAD in the absence of a CTO, although the left ventricular ejection fraction (LV-EF) is not impaired in more than the half of them.[5, 7] Both in patients with preserved and reduced LV-EF the existence of a CTO is an independent risk factor for increased mortality.[7–9] CTOs, in particular those of infarct-related arteries, are associated with a higher burden of ventricular arrhythmias and delivery of therapies from an implantable cardioverter defibrillator (ICD).[8–10] Major adverse cardiac events (MACE) after successful intervention of a CTO ranged between 1.6–3.9%.[11–15] Whereas the rates of the most common MACE such as acute myocardial infarction, cardiac tamponade or coronary vessel perforation were described in detail, until recently, no cases of ventricular arrhythmia due to revascularization of a CTO were reported.[13, 16] Previously, we observed a case of repetitive sustained ventricular tachycardia (VT) that occurred early after a successful CTO intervention in a previously stable patient.[17] While reperfusion arrhythmias (RA) are well known complications after primary PCI for acute myocardial infarction, the prevalence of VT after interventional treatment of CTOs and their clinical significance has not been studied yet. Aim of this registry is to describe the incidence and characteristics of ventricular arrhythmias after PCI of CTOs, as well as to determine possible prognostic factors for their occurrence.

## Methods

All data were derived from a retrospective CTO registry at the Heart Center Leipzig at University of Leipzig. The study protocol conforms to the ethical guidelines of the Declaration of Helsinki. Due to the use of anonymized, retrospectively collected data, no specific ethics vote or individual consent were obtained. CTOs were defined according to the literature as coronary lesions with Thrombolysis in Myocardial Infarction (TIMI) grade 0 flow of at least 3 months duration.[3] CTOs were identified based on the patients’ medical history, previous coronary angiograms showing occlusion of a coronary artery of more than three months duration as well as the morphological characteristics of the lesion. Access was achieved both through antegrade and retrograde approaches. The intervention was performed as previously described.[18, 19]

Between November 2010 and November 2015, a successful PCI of least one CTO, defined as post procedural TIMI grade 3 flow, was achieved in 485 patients. Of them, 342 patients had sufficient follow-up and were included in the analysis. To rule out ventricular arrhythmias as result of acute ischemia prior to PCI, all patients with acute myocardial infarction or cardiogenic shock at initial presentation were excluded. Follow-up data were acquired within routine clinical follow-up. Baseline characteristics, ECG, echocardiographic data, medications including antiarrhythmic drugs and procedural data including the targeted vessel were collected. In cases, in which CTO-PCIs of more than one coronary artery were performed (2.9%), the vessel with the most proximal occlusion was considered the relevant CTO. J-CTO score was used to characterize complexity of CTO interventions. To simplify the analysis, three levels of severity were distinguished (easy with J-CTO score = 0; intermediate with J-CTO score = 1; difficult with J-CTO score≥2) in analogy to similar recent publications.[20, 21] CTOs were defined as CTOs of infarct-related arteries depending on the medical history for myocardial infarction, the presence of pathological Q waves in the corresponding coronary territory or matching scar in cardiac magnetic resonance imaging. After each procedure, vital signs were monitored for at least 6 hours or longer in cases of new or recurrent symptoms. Either new-onset VT or recurrent VT in previously long-term stable patients (defined as freedom from VT for at least one year) was regarded as intervention-associated MACE.

### Statistical analysis

Data structure has been tested on Shapiro-Wilk-test for Gaussian distribution. Dependent on its structure, groups were compared with Mann-Whitney-U-test or t-test. Correlation analysis was performed using the correlation coefficient of Spearman. Univariable odds ratios were calculated using Fisher’s exact probability test. Exact 95% confidence intervals (CI) for these ORs were computed. All clinically relevant variables as well as variables showing statistical significance or a P-value <0.1 in the univariable analysis were included into the multiple logistic regression analysis to create a predictive model for the occurrence of VT after CTO-PCI. A double-sided P ≤0.05 was considered statistically significant. Statistical analysis was performed using the software SigmaPlot 11.0 (Systat Software GmbH, Erkrath, Germany) and SPSS (IBM Deutschland GmbH, Ehningen, Germany).

## Results

### Baseline characteristics

Most enrolled patients were male (81.3%) with a preserved LV-EF (mean 52, standard deviation [SD] 13.1%) and a high-risk profile for CAD. The follow-up period (median 10 months, interquartile range [IQR] 6–16 months) did not differ between patients with post-procedural VTs and those without VT. All baseline data are summarized in Table 1.

**Table 1:** Baseline characteristics.

	*All Patients* *n = 342*	*Patients with VT* *n = 9*	*Patients without VT* *n = 333*	*P-value*
Age [years]	65.2 (SD 11.8)	73.0 (SD 12.3)	65.0 (SD 11.7)	0.05
Sex [% male]	81.3	88.9	81.1	0.55
Prior CABG* [%]	13.3	22.2	13.0	0.29
*Cardiovascular risk factors*				
Diabetes mell. [%]	36.0	33.3	36.0	0.59
Hypertension [%]	95.0	100.0	94.9	0.63
CRF^†^ [%]	18.4	55.6	17.4	0.01
Active Smoking [%]	43.9	22.2	44.7	0.31
BMI^‡^ [kg/m^2^]	27.9 (SD 4.8)	26.8 (SD 3.2)	27.9 (SD 4.8)	0.45
Dyslipidemia [%]	66.1	55.6	66.4	0.49
COPD [%]	7.0	11.1	6.9	0.49
*Symptoms*				
NYHA class baseline	2.0 (IQR 1–3)	3.0 (IQR 2.0–3.5)	2.0 (IQR 1.0–3.0)	0.02
CCS^§^ class baseline	2.0 (IQR 1–3)	2.0 (IQR 1.0–3.0)	2.0 (IQR 1.0–3.0)	0.97
Prior syncope baseline [%]	5.0	22.2	4.5	0.07
NYHA class follow-up	2.0 (IQR 1–2)	2.0 (IQR 2.0–3.0)	2.0 (IQR 1.0–2.0)	0.03
CCS^§^ class follow-up	1.0 (IQR 1–2)	1.0 (IQR 1.0–1.0)	1.0 (IQR 1.0–2.0)	0.17
Syncope follow-up [%]	2.8	11.1	2.6	0.18
*Echocardiography*				
LV EF baseline [%]	52.0 (SD 13.1)	33.1 (SD 5.9)	52.5 (SD 12.9)	<0.001
LV EF follow-up [%]	54.3 (SD 12.4)	37.6 (SD 5.8)	54.7 (SD 12.2)	<0.001
EDV^||^ follow-up [ml]	123.4 (SD 44.9)	181.4 (SD 42.2)	122.0 (SD 44.1)	0.003
*medication*				
Beta-blockers [%]	80.2	88.9	79.9	0.44
ACE^¶^/AT1^#^-antagonists [%]	88.5	88.9	88.4	0.72
Statins [%]	77.5	77.8	77.5	0.67
Diuretics** [%]	12.0	44.4	11.2	0.02
Antiarrhythmics [%]	0.9	11.1	0.6	0.08

* CABG: coronary artery bypass graft

^†^ CRF: chronic renal failure

^‡^ BMI: body mass index

^§^ CCS: Canadian Cardiovascular Society

^||^ EDV: end-diastolic volume

^*¶*^ ACE: Angiotensin converting enzyme

^#^ AT1: Angiotensin II-receptor type 1

** aldosterone antagonist

### Incidence of VT and differences in baseline characteristics

New-onset VT or recurrent VT in previously stable patients were identified in 9 of 342 patients (2.6%) after a successful PCI of a CTO. There were no differences regarding pre-existing comorbidities and CAD risk factors between patients suffering from VT after CTO-PCI and those without VT, with exception of chronic kidney disease (OR 5.93, 95% confidence interval 1.54–22.7, P<0.001). Patients in the VT-group were significantly older and had an impaired LV-EF in comparison to patients without VT. Heart failure, measured as New York Heart Association (NYHA) class, was more severe in the VT-group. Both NYHA class and the LV-EF remained significantly worse in VT patients by the end of the follow-up period. Because of the generally normal LV-EF in our population, only 17 patients (5%) had a previously implanted ICD. Due to the impaired LV-EF, ICDs were significantly more present in the VT-group (66.6% versus 3.3%, P<0.001). The majority of patients in both groups were treated with beta-blockers, angiotensin converting enzyme-inhibitors/angiotensin II receptor type 1-blockers and statins. Aldosterone antagonists were prescribed more often in the VT-group. All baseline data are summarized in Table 1.

### Procedural data

Most of the study participants had multivessel CAD (258 patients, 75.4%). Evidence of ischemia (exercise stress ECG, dobutamine stress echocardiography or cardiac stress magnetic resonance imaging) was demonstrated in 172 patients (50.3%) before PCI. The average fluoroscopy time was 14.0 minutes (SD 12.6 minutes) and a mean of 232.2 ml (SD 119.3 ml) of contrast agent was used. Target vessel of the CTO-PCI was the left anterior descending coronary artery in 100 cases (29.2%), of them in the proximal segment in 20 cases (5.8%). The right coronary artery was opened in 179 patients (52.3%), the left circumflex artery in 61 patients (17.8%) and bypass grafts in 2 patients (0.6%), respectively. Data regarding the J-CTO score were available in 332 of 342 patients (97.1%). There were no technical differences in the procedure between patients with and without VT which was reflected in a comparable distribution of the modified J-CTO score in the 323 patients with available severity leveling (no VT vs. VT: easy in 10.8% vs. 11.1%, intermediate in 32.8% vs. 44.1%, difficult in 56.3% vs. 44.4%, p = 0.75). CTO-PCI of a previous infarct-related artery was performed in 118 patients (34.5%). In one patient in the VT-group, there were clinical and ECG signs of procedure-related myocardial infarction. This patient underwent a second urgent revascularization but continued to suffer from recurrent VTs afterwards.

### Time of occurrence and characteristics of the VT

Ventricular arrhythmias after CTO-PCI were observed in 9 study participants (2.6%). In all cases, monomorphic VTs with cycle length ranging from 400 to 430 milliseconds were recorded. The mean time from CTO-PCI to hospital discharge was 2.7 days (SD 5.7 days). Detection of VT led to a significant prolongation of the hospital stay with a time to discharge of 7.2 days (SD 7.1 days) in the VT-group versus 2.4 days (SD 4.5 days) in the non-VT-group (P<0.001). Two patients needed electrophysiological study and radiofrequency catheter ablation because of refractory VT. The median time from the CTO-PCI to the first occurrence of VT was 1 day (IQR: 0.25–4.75 days). Of 9 patients with VT, five patients had first documentation of VT and four patients had a previous history of ventricular arrhythmias but were free from ventricular arrhythmia for at least one year before the intervention. Time to new-onset VT was shorter than the time to VT-recurrence in patients with previously known VT without a statistically significant difference (2.5 [SD 2.4] vs. 4.7 [SD 4.0] days, P = 0.297).

### Predictors for VT-occurrence

Univariable analysis was performed for VT occurrence as a dependent variable. Age (R^2^ = 0.11, P = 0.04), chronic kidney disease (R^2^ = 0.16, P = 0.004), LV-EF at enrollment (R^2^ = 0.24, P<0.001), NYHA class at enrollment (R^2^ = 0.13, P = 0.02) and the intervention of a CTO of an infarct-related artery (R^2^ = 0.15, P = 0.006) were associated with occurrence of VT after CTO-PCI. Furthermore, treatment with aldosterone antagonists (R^2^ = 0.16, p = 0.003) and the presence of an ICD (R^2^ = 0.45, P<0.001) were predictors for VT after CTO-PCI. Neither cardiovascular risk factors nor the existence of multivessel CAD were predictive for the onset of VT after CTO-PCI. The targeted vessel was not associated with occurrence of VT. Including the above-mentioned variables into the multiple logistic regression analysis, LV-EF (OR 0.91, 95% confidence interval 0.84–0.98, P = 0.01) was the only variable significantly associated with the likelihood for post-procedural occurrence of VT.

### Mortality

During follow-up, death occurred in 12 patients (3.5%) with one death in the VT-group (11.1%) compared to 11 deaths in the non-VT-group (3.3%) without a statistically significant difference between both groups (P = 0.24). Two patients in the non-VT-group died during the initial hospital stay because of mesenteric ischemia and coronary artery perforation with hemothorax, resulting in an in-hospital mortality of 0.6%. None of the deaths during the follow-up was arrhythmia-associated.

## Discussion

Ventricular arrhythmias as a consequence of CTO-PCI have not been investigated systematically. Previously, we reported a case of refractory reperfusion-associated ventricular arrhythmia occurring early after CTO-PCI necessitating catheter ablation.[17] In this registry, we sought to determine the incidence and the characteristics of new-onset VT as possible RAs in patients with successfully opened CTOs.

During follow-up the observed incidence of post-procedural VT early after CTO-PCI was relatively low (2.6%) but caused a significant prolongation of the hospital stay. To the best of our knowledge, in larger registries and meta-analyses, data regarding VT occurrence after revascularization of CTOs are lacking. Importantly, patients included in our registry share similar baseline characteristics with cohorts in previously published CTO-studies.[15, 22] Consistent with previous CTO registries, most patients in our study had preserved LV-EF, multi-vessel CAD, and the most frequently targeted vessels were the RCA and the LAD.[15, 22, 23] In contrast to RAs in patients with acute myocardial infarction that are mostly observed within minutes to one hour after the onset of reperfusion, most of the VTs in patients with CTO occurred later after the intervention and were observed as late as 9 days after CTO-PCI.[24] While the cycle length of RAs in STEMI usually does not exceed 500 milliseconds, the VTs after CTO-PCI were monomorphic and faster with a cycle length about 400 milliseconds. However, those arrhythmias are still slower compared to monomorphic VTs in ischemic cardiomyopathy in general.[17, 25] Furthermore, monomorphic VTs have been infrequently reported in the settings of acute ischemia, while an accelerated idioventricular rhythm or frequent premature ventricular complexes were more frequently observed as early RAs.[24, 26]

An enhanced cellular automaticity and triggered activity caused by changes in the intracellular concentration of potassium, sodium, magnesium and free oxygen radicals are regarded responsible for reperfusion-associated ventricular arrhythmias in STEMI patients.[27, 28] This could be a possible mechanism for the single patient that experienced VT after acute stent thrombosis in the early post-PVI period. However, the peculiar characteristics of VTs after successful CTO revascularization suggest different electrophysiological mechanisms from that of RAs after acute myocardial infarction. Hibernating myocardium is a highly vulnerable substrate susceptible for arrhythmias due to changes in cellular metabolism caused by recurrent or chronic ischemia.[29] There are experimental evidences that prolonged chronic ischemia can lead to denervation of hibernated but viable myocardium.[30] Apart from structural changes and fibrosis, the denervation-associated hypersensitivity may increase the susceptibility to ventricular arrhythmia in patients with CTO. A study using signal-averaged electrocardiography found abnormal late potentials to be more frequent in patients with CTO compared to healthy controls.[31] In a recent retrospective analysis of patients ablated for VT, the electro-anatomical voltage maps of patients with CTO showed a larger area of dense scar with electrograms <0.5 mV and a larger border zone with pathological signals compared to non-CTO patients. Moreover, patients with CTO had significantly higher rates of VT-recurrence after catheter ablation in comparison to non-CTO counterparts.[32] Nombela-Franco et al. showed an association between CTOs and an increased number of adequate ICD-therapies.[9] Other trials confirmed these findings, in which especially CTOs of infarct-related arteries were predictive for a higher number of VTs.[8, 10] Interestingly, in our cohort the majority of sustained VTs also occurred in patients with revascularization of infarct-related arteries, whereas the targeted vessel was not related the occurrence of ventricular arrhythmia. This is in line with the findings of Di Marco et al. considering CTOs of infarct-related arteries as an independent risk factor for VT.[8, 32] However, in the multivariable model of our cohort the presence of such CTO was not associated with the occurrence of VT and the only relevant predictor for VT was impaired LV-EF.

RAs in the setting of STEMI are considered to be benign as some studies even suggested a reduced mortality in patients with RAs.[33] On the other hand, there are publications suggesting an association of RA-occurrence with a larger infarct expansion and a lower LV-EF.[34] Although CTOs usually are considered to convey a worse prognosis both in patients with stable CAD and acute myocardial infarction, the occurrence of VT after CTO-PCI was not associated with an increased mortality and mortality rates were comparable to other studies.[7, 12, 14] We observed only one death among patients with VTs after CTO-PCI which was not related to VT. However, in order to detect potentially lethal VTs, an extended in-hospital monitoring of all patients for at least 24 hours after the intervention as well as a 7-day-Holter-monitoring after discharge for all patients with reduced LV EF may be recommended.

## Limitations

This study is not a randomized trial. Because of the retrospective nature of the study it was not possible to identify the causes of those VTs occurring before the CTO intervention. Acute cardiac ischemia during the complex and prolonged intervention may also cause ventricular arrhythmia. A causal relationship between revascularization and occurrence of VT cannot be deduced from the available data. However, an association is highly likely due to the long period of time without arrhythmia recurrences before the intervention and the close temporal relationship of VT-occurrence and the CTO-PCI. Due to the retrospective data collection, some information relevant to the interpretation of the results is missing, such as procedural data. The occurrence of asymptomatic non-sustained ventricular arrhythmia outside the initial monitoring period of 6 hours or after discharge may lead to underestimation of the prevalence of ventricular arrhythmia in this population. However, sustained VTs usually urge the patients to seek medical help and are hardly to remain undetected.

## Conclusions

VT can infrequently occur soon after CTO-PCI as a possible RA. In comparison to RAs after acute myocardial infarction, these are more rapid, monomorphic and occur later after the intervention. Impaired LV-EF was the only independent predictor for the occurrence of VT after CTO-PCI. Although those VTs seemed not to influence mortality, it resulted in significant prolongation of the hospital stay. Awareness of this possible complication is required and longer, systematic rhythm monitoring of these patients may be recommended.

## Perspectives

Considering the results of this study, a more detailed investigation of ventricular arrhythmias related to CTO-PCIs should be performed. Because of the overall low event rate, the inclusion of an even larger number of patients should be considered. The comparison with non-successful CTO-PCIs and the additional correlation with morphological information especially from cardiac magnetic resonance imaging would allow a better work-up of possible risk factors as well as possible mechanisms for the development of VTs in this context.

## Supporting information

S1 File Raw data. (XLSX)

S1 Table Event rate of ventricular arrhythmias after CTO-PCI in different subgroups of patients. (DOCX)

## Abbreviations

CAD: coronary artery disease
CTO: chronic total occlusion of a coronary artery
LV-EF: left ventricular ejection fraction
MACE: major adverse cardiac event
NYHA: New York Heart Association
PCI: percutaneous coronary intervention
RA: reperfusion arrhythmia
TIMI: Thrombolysis in Myocardial Infarction
VT: ventricular tachycardia
