# Supplementary material for: Incidence and characteristics of ventricular tachycardia in patients after percutaneous coronary revascularization of chronic total occlusions
Source: PLoS One. 2019 Nov 22;14(11):e0225580. doi: 10.1371/journal.pone.0225580 (PMC6874319; doi:10.1371/journal.pone.0225580)
Supplement: S1 Table — (DOCX) [file pone.0225580.s002.docx]

**Supplemental material**

Supplemental Table 1: event rates of ventricular arrhythmias after CTO-PCI in different subgroups of patients

| Event rates and Univariable Analysis total study cohort | | | |
| --- | --- | --- | --- |
| Variable | post-procedural ventricular arrhythmia | | P-value |
|  | % (n/N) | OR (95% CI) |  |
| Gender  male  female | 1.6 (1/64)  2.9 (8/270) | 1.87 (0.23-15.2) | 1.00 |
| Diabetes mellitus  no  yes | 2.7 (6/219)  2.4 (3/123) | 0.89 (0.22-3.61) | 1.00 |
| Chronic kidney disease  no  yes | 1.4 (4/279)  7.9 (5/63) | 5.93 (1.54-22.7) | 0.01 |
| Dyslipidemia  no  yes | 3.4 (4/116)  2.2 (5/226) | 0.63 (0.17-2.41) | 0.49 |
